# Eliminating *Plasmodium falciparum* in Hainan, China: a study on the use of behavioural change communication intervention to promote malaria prevention in mountain worker populations

**DOI:** 10.1186/1475-2875-13-273

**Published:** 2014-07-13

**Authors:** Chang-hua He, Xi-min Hu, Guang-ze Wang, Wei Zhao, Ding-wei Sun, Yu-chun Li, Chun-xiang Chen, Jian-wei Du, Shan-qing Wang

**Affiliations:** 1 Hainan Provincial Center for Disease Control and Prevention, No.44, Haifu Road, 570203 Haikou, Hainan province, China

## Abstract

**Background:**

In the island of Hainan, the great majority of malaria cases occur in mountain worker populations. Using the behavioral change communication (BCC) strategy, an interventional study was conducted to promote mountain worker malaria prevention at a test site. This study found the methods and measures that are suitable for malaria prevention among mountain worker populations.

**Methods:**

During the *Plasmodium falciparum* elimination stage in Hainan, a representative sampling method was used to establish testing and control sites in areas of Hainan that were both affected by malaria and had a relatively high density of mountain workers. Two different methods were used: a BCC strategy and a conventional strategy as a control. Before and after the intervention, house visits, core group discussions, and structural surveys were utilized to collect qualitative and quantitative data regarding mountain worker populations (including knowledge, attitudes, and practices [KAPs]; infection status; and serological data), and these data from the testing and control areas were compared to evaluate the effectiveness of BCC strategies in the prevention of malaria.

**Results:**

In the BCC malaria prevention strategy testing areas, the accuracy rates of malaria-related KAP were significantly improved among mountain worker populations. The accuracy rates in the 3 aspects of malaria-related KAP increased from 37.73%, 37.00%, and 43.04% to 89.01%, 91.53%, and 92.25%, respectively. The changes in all 3 aspects of KAP were statistically significant (*p* < 0.01). In the control sites, the changes in the indices were not as marked as in the testing areas, and the change was not statistically significant (*p* > 0.05). Furthermore, in the testing areas, both the percentage testing positive in the serum malaria indirect fluorescent antibody test (IFAT) and the number of people inflicted decreased more significantly than in the control sites (*p* < 0.01).

**Conclusion:**

The use of the BCC strategy significantly improved the ability of mountain workers in Hainan to avoid malarial infection. Educational and promotional materials and measures were developed and selected in the process, and hands-on experience was gained that will help achieve the goal of total malaria elimination in Hainan.

## Background

The island of Hainan is the southernmost island and province in China. It is situated between latitudes 18°10’ N and 20°10’ N and longitudes 108°37’ E and 111°03’ E. Hainan Island is a tropical area with a climate and environment ideally suited for the breeding of the mosquito varieties *Anopheles minimus* and *Anopheles dirus*, both vectors of which are malaria transmission. Malaria is actively transmitted throughout the year in Hainan, and historical data show that the island was once the site of the most devastating malaria epidemics in China, especially of the *P. falciparum* strain. The *Anopheles minimus* distribution covers almost the entire island; in 1955, the percentage of residents in the south-central mountain region carrying the parasite was over 50%, and the disease rate was as high as 1,036.6/10,000 [1-3].

Based on estimates by the WHO, the global rate of malaria deaths decreased by 26% between the years 2000 and 2010. However, in 2010, there were still 219 million people infected, resulting in 660,000 deaths that year [4]. In China, due to the unwavering efforts of the government and all levels of malaria prevention personnel, malaria epidemics throughout China have also declined substantially in the past 50 years [5]. Although China and Hainan Island have made good progress in the fight against malaria, in the previous malaria-control strategies, there was a lack of recognition, understanding, and application of BCC strategies [6-8]. Hainan Island has 50 years of malaria control records without any systematic research regarding BCC strategies, and there is a similar absence of literature regarding this strategy in other parts of China [3,9,10]. In contrast, in an earlier malaria control stage, researchers conducted an intervention study in parts of Hainan Island and obtained good results in the research stage; however, these intervention measures encountered a lack of persistence or long-term acceptance rates. Therefore, these intervention measures have not been widely implemented, especially given the overall decline of malaria cases in Hainan [10-14].

According to WHO recommendations, when the malaria incidence rates in a country or region decrease to a very low level, it is necessary to conduct official efforts to eradicate malaria [15]. Considering this guideline, in May 2010, 13 Chinese government departments jointly developed and released a plan to eliminate malaria in most of the country by 2015 and to achieve the goal of national malaria elimination by 2020 [16]. Hainan has effectively reduced the incidence and mortality of malaria, and malaria-endemic areas continue to shrink [17-19]. In recent years, especially since 2003, malaria control in Hainan has received strong support from the Chinese government and the first and fifth rounds of the Global Fund Malaria Project [20], greatly enhancing the ability to control the spread of malaria in Hainan Island and reducing local malaria epidemic levels to their lowest point in history. Hainan has two kinds of malaria (*Plasmodium falciparum* and *Plasmodium vivax*) and, according to 2012 Chinese reports on infectious diseases, Hainan Island has gone through three consecutive years without any reports of local *P. falciparum* malaria cases. By the standards of the World Health Organization, Hainan Province has achieved the elimination of *P. falciparum* malaria [5,21].

In the mountain areas of Hainan, most residents make a living by farming, and historical data show that most cases of malaria infection in Hainan have their source in populations that live or work is at high risk for malaria infection in the mountains [22-24]. The vast majority of these people are planters of betel nuts and rubber, and in the process of agricultural development, they inevitably must stay overnight in the mountains. They mostly live in temporary dormitories close to *Anopheles* breeding sites. These living conditions are poor, and the workers are scattered; consequently, measures to control mosquitoes and to diagnose and treat malaria in a timely manner are difficult to implement, which is a major problem confronting anti-malaria interventions. Ensuring that malaria prevention is effectively conducted among mountain worker populations will play a decisive role in achieving the future elimination of *P. falciparum* and *Plasmodium vivax* malaria in Hainan Island.

To eliminate malaria, residents in the target area must have a high level of knowledge regarding the transmission and prevention of malaria and must maintain a high level of participation [16,25-29]. Behavioural change communication (BCC) is a behaviour-shaping or -changing measure that mainly aims to use information education communication (IEC) to improve the knowledge level of the population, create a supportive social environment that is conducive to forming target behaviours, and provide material and moral support to help overcome factors that would affect target behaviours [25,30]. Using BCC strategies to promote healthy behaviours against malaria is based on verified malaria control theories and behaviour change models and examples. Through the development of appropriate actions and information that are acceptable to the community - taking advantage of exchanges within the community to spread related information and behaviour - and the honest exchange of dialogue, the attitude and participation of residents in malaria prevention and treatment can be improved; the end goal is to promote positive behaviour, build community participation, and create a strong prevention system [31,32].

Between March 2009 and December 2012, the researchers selected six towns (farms) with concentrated mountain worker populations in the south-central mountain areas of Hainan to conduct research trials on using BCC intervention strategies for the promotion of malaria prevention. By analysing malaria-related KAPs and changes in serum markers before and after the intervention, the authors explored and accumulated methods to promote malaria prevention in mountain worker populations using the BCC strategy and thereby how to accelerate the progress of malaria elimination in Hainan.

## Methods

### Sampling of mountain worker populations

**Figure 1:**
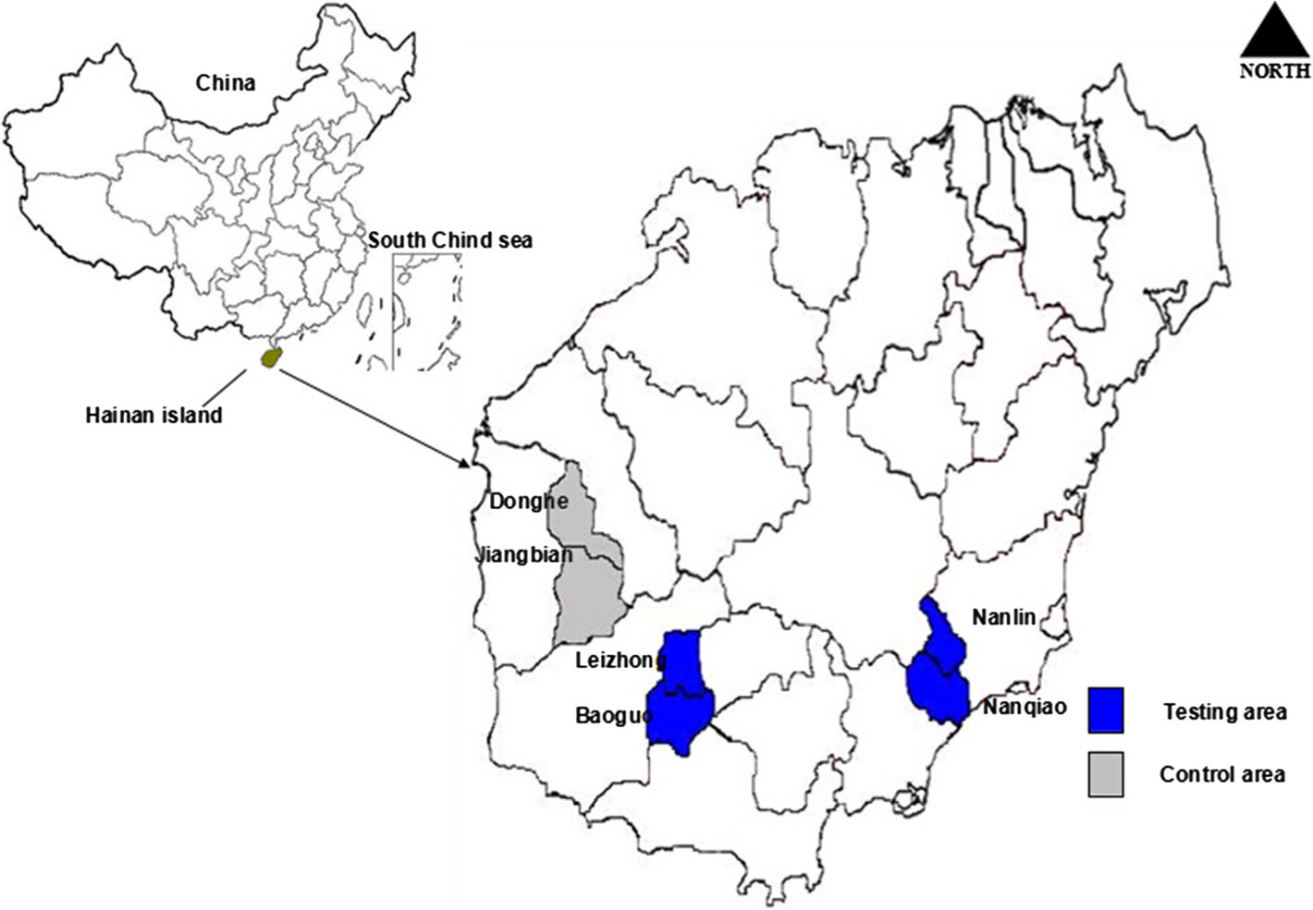
Map of China and Hainan island and intervention classification area.

Among the towns and cities in Hainan that were malaria-endemic and had higher concentrations of mountain workers, representative sampling methods were used to arrange the cities, towns, and farms based on their malaria incidence rates in 2007–2008. One farm and one town with high rates of malaria and large numbers of mountain workers were chosen to be surveyed. Wanning (Nanlin farm and Nanqiao town) and Ledong (Lezhong farm and Baoguo farm) were chosen as testing areas, while the Dongfang region (Donghe town and Jiangbian town) was chosen as a control site (Figure 1). The areas were all greater than 100 km apart, and the residents of each area were largely isolated from those of other areas. Surveys included all mountain workers within the towns or farms that were at least 15 years of age and had lived in the area for at least three years. At the time of the final survey, 100 mountain workers were randomly selected from the testing areas to survey their feedback on BCC intervention material.

### Malaria serum samples and control (positive and negative) grouping

The inclusion criteria were as follows: (1) individuals who had lived in the selected mountain areas for ≥ three years or who had worked in the mountains for ≥ three years, including local and migrant workers; and (2) individuals with titers ≥ 1:20 in number when tested with IFAT based on malaria serological survey methods [33]. Workers who had entered the mountains recently or occasionally, who did not reside continuously in the selected regions, or who were under 15 years of age were excluded.

### Development of the BCC intervention material

**Figure 2:**
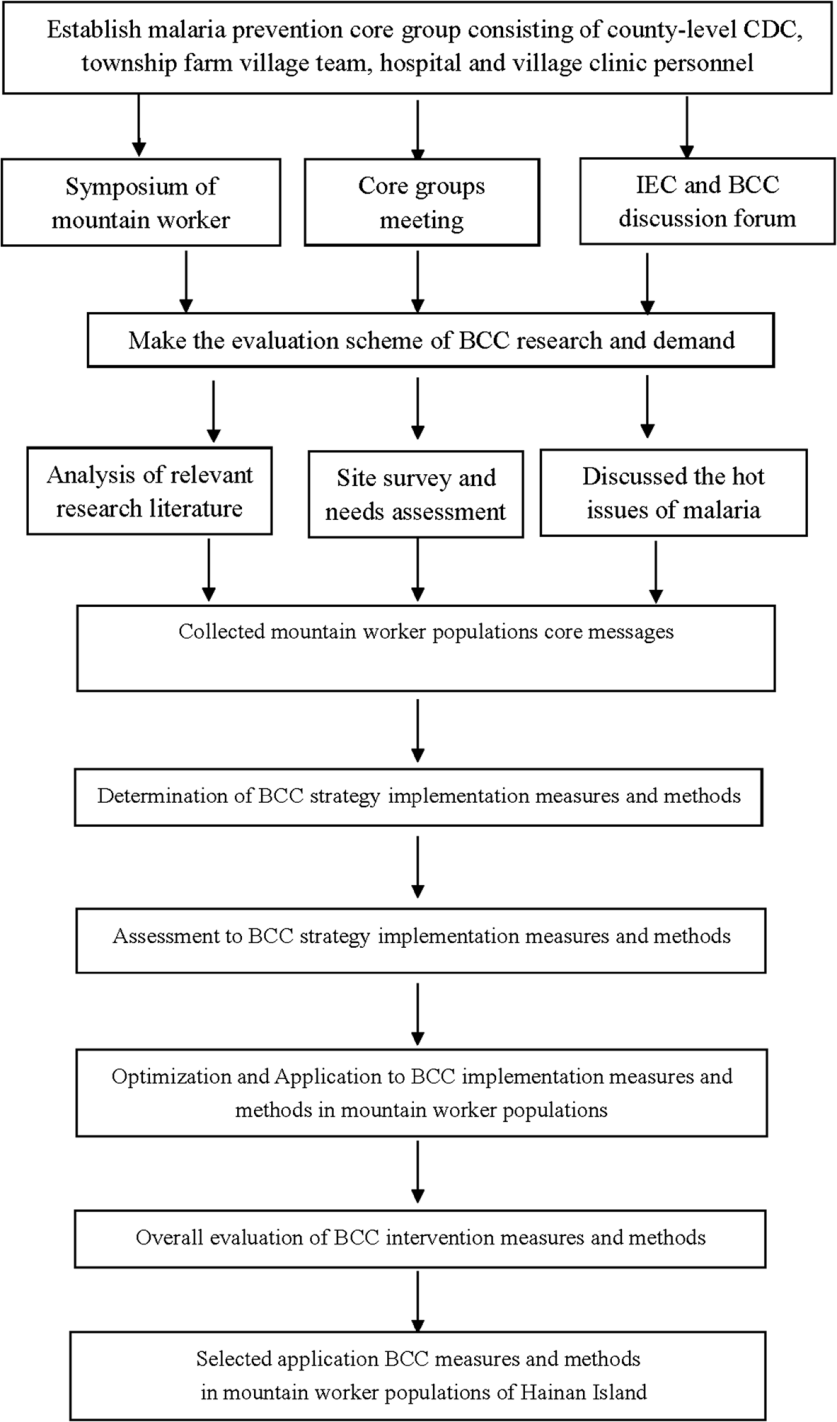
Schematic diagram for development of BCC intervention material.

After a needs evaluation for malaria-related IEC and BCC in Hainan’s mountain worker populations [25], and after referencing domestic and foreign educational materials and methods (especially those of Hainan), the interventional methods and educational materials were designed for this study, using the following steps (Figure 2).

### Techniques for disseminating the interventional information

Based on the needs evaluation, communication materials were chosen that were practical and would likely be favored by mountain workers (such as straw hats, slogan, towels, fans, calendars, medicine bags, folding leaflets, signs, public network, posters, and T-shirts), with more than 10 forms of media overall. A public health website and video compact disc (VCD) materials were created taking into account local customs and ethnic and linguistic characteristics.

### Intervention methods

During the period between October 2009 and October 2012, only passive anti-malarial measures such as free diagnosis and treatment, early treatment, and indoor residual spraying were performed in the control areas. In the testing areas, besides the routine measures, the following measures were conducted: (i) the establishment of malaria prevention core groups consisting mainly of town farm teams, health institutions, town health and village clinic personnel, and strengthening of community promotion and participation, with a system of incentives for discovering malaria on a regular basis; (ii) the distribution of long-lasting insecticide-treated nets (LLINs) and insecticide-treated nets (ITNs), which were treated twice a year in the spring and fall, to the residents, and the provision of indoor residual spraying services; (iii) needs assessments for KAP baseline indicators and the consequent development of a comprehensive model of malaria-related BCC policy interventions (seminars, lectures, leaflets, posters, audio-visual medias, etc.) for educational and promotional purposes, with a frequency of 2–3 times/year; (iv) using the public health network Liquid Crystal Display (LCD) digital media platforms built by the Hainan government in health provider sites, community health service stations, urban and rural hospitals, pharmacies and other public places, malaria-related health information based primarily on the needs of the mountain worker populations was put on rolling display every day, in both the Hainan dialect and Mandarin.

The core messages in the interventional media and promotional materials were the following: (1) mosquitoes bite people and spread malaria; (2) if you experience chills and fever, get urgent medical attention; (3) anti-malarial medication is free, and the therapy must be thorough; (4) malaria is a preventable and treatable parasitic disease; (5) eliminating the hazards of malaria is the honor of this generation and will benefit future generations; (6) early diagnosis and treatment of malaria is essential; (7) for the ecological management of mosquito breeding sites in residential areas, employ reasonable indoor residual spraying, ITNs, and LLINs. The media messages included voices both in Mandarin and in the Hainan dialect.

### Survey information collection

After reviewing the historical data, qualitative and quantitative surveys were designed, which were subjected to reviews by specialists, pre-investigated, and then modified. The malaria-related KAP questionnaire concerned the following: (1) general demographic characteristics: gender, age, ethnicity, commonly used language, education level and other demographic characteristics; (2) mosquito control- and prevention-related basic knowledge; (3) malaria control- and prevention-related basic knowledge; (4) malaria control- and prevention-related basic attitudes; (5) malaria prevention- and treatment-related practices and behaviours, including the type of work in the mountains; living conditions and use of bed nets in the mountains; frequency of overnight stays in the mountains; use of preventative medication before overnight stays in the mountains; whether the subject received malaria-prevention education, used a bed net at home, accepted ITNs, or actively used bed nets in the mountains; whether the subject was taking preventive measures during overnight stays in the mountains (such as bed nets, insect repellents, making a fire or spraying insecticide around the temporary dormitories); whether the subject habitually slept outdoors; whether the subject was aware of the symptoms of malaria; and so on. There were five aspects and 40 problems for the malaria control and prevention-related KAPs. After the intervention, 100 randomly selected mountain workers from the testing areas were asked whether they could recall the various promotional materials and their core messages, as well as the degree to which they liked these materials, to survey their feedback regarding the BCC intervention material. The investigators were mainly public health physicians from county-level CDC and local hospitals in the testing regions, with a total number of 18, and they were all trained together by 4 specialists from the Hainan Provincial CDC. The interviews took the form of in-home, one-on-one, face-to-face visits; upon completion of the questionnaire, blood was collected from the finger or earlobe, placed on filter paper, and coated on a microscopic slide. During March-April 2009 and November-December 2012, mountain workers in the test area and control area were tested for malaria-related KAP, infection, serum markers, and blood parasite rate, and these data were used as the baseline and endpoint of the investigation.

### Malaria infection data and blood tests

The Chinese Infectious Disease Network reporting system was used to track the number of people infected with malaria in the testing and control sites. Two local qualified professionals performed the microscopic examinations for each area.

### Malaria serum antibody tests

The subjects’ earlobe or fingertip blood was collected and dripped into a circle on filter paper (20 μL of blood fills the circle), with 1 round drop of blood per filter paper. The blood samples were allowed to dry naturally and were then stored in a plastic bag with a drying agent at -20°C. Blood samples were sent to the Hainan Province Center for Disease Control and Prevention’s National Malaria Laboratory, where they were subjected to a serum Indirect Fluorescent Antibody Test (IFAT) in accordance with the “Manual of Malaria Control” [33]. Antibody titers ≥ 1:20 were regarded as positive for malaria antibodies. *Plasmodium cynomolgi* antigen from cynomolgus macaques was used for detection [34], provided by the Chinese Center for Disease Control and Prevention’s Institute of Parasitic Diseases.

### Malaria prevention knowledge and attitudes survey

Answering at least six questions correctly was considered passing. The scoring criteria were as follows: (1) answer deemed correct, 4 points; (2) answer deemed basically correct, 2 points; (3) answers that did not meet the standards or were lacking were deemed incorrect, 0 points. Scores ≥ 24 points were considered passing grades, while those < 24 were considered failing grades.

### Malaria prevention practices survey

The survey included questions such as whether the subject had the habit of sleeping outdoors, whether mosquito prevention measures were taken (bed nets, mosquito incense, or mosquito repellant), willingness to take a blood test upon getting a fever, and whether insecticide-treated bed nets were used, etc., with 10 questions in total. Eight good behaviours constituted a passing grade. The scoring criteria were: (1) answer deemed correct, 4 points; (2) answer deemed basically correct, 2 points; (3) answers that did not meet the standards or were lacking were deemed incorrect, 0 points. Scores ≥ 32 points were considered passing grades, while those < 32 were considered failing grades.

After the surveys were collected and organized, two people independently entered the data using EpiData 3.1 software, and they were compared to ensure they were identical and logically consistent. The data were then imported into SPSS 17.0 for statistical analysis, with a statistical significance level of α = 0.05.

## Results

### General information

Before the BCC intervention strategy, there were 40,746 people in the testing area and 22,875 people in the control area in 2009, and the surveyed mountain worker population totaled 925 people; this number included 546 people in the testing area and 379 in the control area, with mean ages of (41.40 ± 9.82) years and (39.84 ± 10.30) years, respectively. There were 495 people of the Li ethnicity in the testing area, and 379 people in the control area usually communicated in the Hainan dialect. After the intervention, there were 41,244 people in the testing area and 23,741 people in the control area; in 2012, the surveyed population totaled 948 people, with 555 in the testing area and 393 in the control area, and the mean ages were (45.41 ± 10.40) years and (47.08 ± 11.33) years, respectively. There were 523 people of the Li ethnicity in the testing area, and 388 people in the control area usually communicated in the Hainan dialect. There were no statistically significant differences between the mountain worker populations of the testing and control areas in terms of gender, mean age, or educational level, both before and after the BCC strategy intervention; thus, the 2 populations were comparable.

### Malaria-related KAP survey

The results show that before the BCC intervention strategies, the accuracy rates of malaria-related KAP knowledge and proper behaviour were low in the mountain worker populations of both the testing and control areas. In all three aspects, regardless of gender, age group, or education level, no group exceeded 50% in terms of knowledge or correct behaviour (Table 1). Table 2 compares the results of the test area and the control area before intervention.

**Table 1:** Baseline of accuracy rate of malaria related KAP among mountain worker populations

**Content**	**Group**	**Testing area**				**Control area**			
		**Population**	**K (%)**	**A (%)**	**P (%)**	**Population**	**K (%)**	**A (%)**	**P (%)**
Gender	Male	316	121 (38.29)	112 (35.44)	140 (44.3)	181	84 (46.41)	75 (41.44)	81 (44.75)
	Female	230	85 (36.96)	90 (39.13)	95 (41.30)	198	69 (34.85)	74 (37.37)	87 (43.94)
Age group (years)	< 20	47	20 (42.55)	22 (46.81)	19 (40.43)	38	18 (47.37)	18 (47.37)	15 (39.47)
	20-40	325	103 (31.69)	115 (35.38)	132 (40.62)	264	99 (37.50)	101 (38.26)	113 (42.80)
Educational status	> 40	174	83 (47.70)	65 (37.36)	84 (48.28)	77	36 (46.75)	30 (38.96)	40 (51.95)
	Illiteracy/Semiliterate	38	10 (26.32)	9 (23.68)	17 (44.74)	121	39 (32.23)	42 34.71)	48 (39.67)
	Primary education	105	33 (31.43)	35 (33.33)	47 (44.76)	225	101 (44.89)	90 (40.00)	100 (44.44)
	Junior high school and above	403	163 (40.45)	158 (39.21)	171 (42.43)	33	13 (39.39)	17 (51.52)	20 (60.61)
Sum		546	206 (37.73)	202 (37.00)	235 (43.04)	379	153 (40.37)	149 (39.31)	168 (44.33)

K = Rate of malaria knowledge; A = Rate of correct attitude; P = Rate of good practice.

**Table 2:** The proper rate of the major indexes of malaria-related KAPs among mountain worker populations before intervention

**Index**	**Testing area**		**Control area**		***χ***^**2**^	***p***
	**n**	**%**	**n**	**%**		
How malaria is spread	205	37.60	167	44.10	3.95	0.05*
Onset symptoms of malaria	314	57.60	199	52.50	2.27	0.13
Types of malaria	88	16.10	74	19.50	1.80	0.18
Malaria prevention methods	190	34.80	112	29.60	2.80	0.09
Mosquito breeding grounds	236	43.20	150	39.50	1.22	0.27
Anti-mosquito effect of insecticide treated bed nets	210	38.50	164	43.20	2.15	0.14
Whether avoiding mosquito can prevent malaria	264	48.30	209	55.10	4.13	0.04*
Using bed nets actively in the mountains	68	12.50	53	14.00	0.46	0.50
Acceptance of information about malaria	346	63.30	225	59.40	1.52	0.22
Preventive drugs for malaria	106	19.50	78	20.70	0.19	0.66
Treatment drugs for malaria	216	39.50	141	37.10	0.53	0.47
Method of preventive drugs	53	9.70	41	10.90	0.30	0.58
Having a plan to prevent mosquitoes	225	41.30	165	43.50	0.50	0.48
Having a plan to not sleep outdoors in the mountains	239	43.70	143	37.60	3.37	0.07
Taking preventive measures while working in the mountains	209	38.20	165	43.60	2.57	0.11
Percentage sleeping outdoors in the mountains	179	32.78	136	35.90	0.96	0.33
Actual use of a bed net at home	128	23.44	104	27.50	1.90	0.17
Accepting a blood test when having a fever	238	43.59	206	54.30	10.38	0.00*
Accepting ITNs at home	187	34.25	144	37.90	1.37	0.24
What to do when suffering from malaria	327	59.80	233	61.40	0.24	0.63

*p < 0.05.

Nine hundred twenty-five persons (testing area, 546; control area, 379) participated in the survey before undergoing the BBC-strategy-intervention.

### Serum malaria IFAT and baseline parasite-infection rate

Malaria serology results from the testing and control areas are shown in Table 3. Aside from the data shown, in 2008, there were 10 cases of malaria infection reported in the area, including seven in the testing area and three in the control area.

**Table 3:** Results of the IFAT and blood smear test in the testing and control areas before intervention

**Group**	**Population**	**IFAT number testing positive (%)**	**GMRT**		**Positive diagnosis with microscopic examination**	
			**Positive**	**Total**	**Population**	**%**
Testing area	546	22 (4.03)	27.41	10.42	1	0.18
Control area	379	24 (6.33)	25.94	10.62	1	0.26

IFAT = Indirect fluorescent antibody test. GMRT = Geometric mean reciprocal titer. IFAT Positive: ≥ 1:20.

### Effectiveness of the BCC intervention strategy

**Figure 3:**
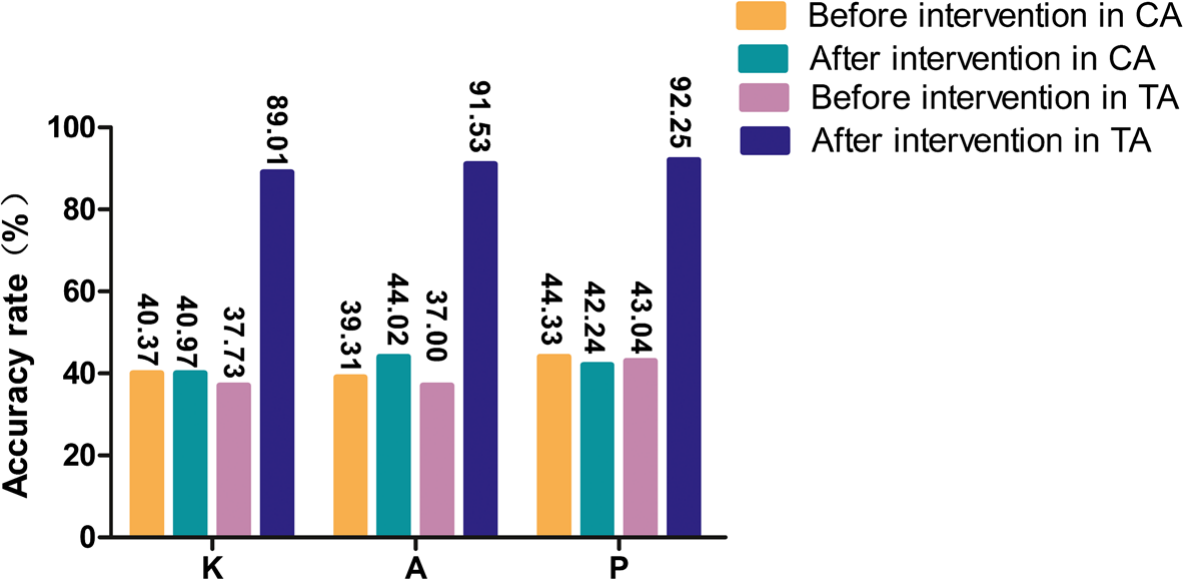
**Comparison of changes in malaria-related KAP accuracy rate between testing and control area.** *CA: control area; TA: testing area.

Based on changes in the malaria-related KAP in mountain workers in the test and control areas after BCC intervention (Table 4), there was a greater change in knowledge, attitudes and practices in the test area compared with the control area, and this difference is statistically significant (*p* < 0.01). Figure 3 shows a comparison of changes in the malaria-related KAP scores’ accuracy rate between the test area and the control area.

**Table 4:** Change in the accuracy rate of the malaria related KAP among mountain worker populations after intervention

**Content**	**Group**	**Testing area**				**Control area**			
		**Population**	**K (%)**	**A (%)**	**P (%)**	**Population**	**K (%)**	**A (%)**	**P (%)**
Gender	Male	295	249 (84.41)	257 (87.12)	263 (89.15)	201	91 (45.27)	93 (46.27)	84 (41.79)
	Female	260	245 (94.23)	251 (96.54)	249 (95.77)	192	70 (36.46)	80 (41.67)	82 (42.71)
Age group (years)	<20	58	54 (93.10)	51 (87.93)	47 (81.03)	45	25 (55.56)	22 (48.89)	15 (33.33)
	20-40	306	275 (89.87)	291 (95.10)	285 (93.14)	237	83 (35.02)	103 (43.46)	98 (41.35)
	>40	191	165 (86.39)	166 (86.91)	180 (94.24)	111	53 (47.75)	48 (43.24)	53 (47.75)
Educational status	Illiterate/semiliterate	47	43 (91.49)	40 (85.11)	43 (91.49)	115	42 (36.52)	5 (44.35)	48 (41.74)
	Primary education	121	107 (88.43)	104 (85.95)	111 (91.74)	231	102 (44.16)	99 (42.86)	93 (40.26)
	Junior high school and above	387	344 (88.89)	364 (94.06)	358 (92.51)	47	17 (36.17)	23 (48.94)	25 (53.19)
Total		555	249 (89.01)	508 (91.53)	512 (92.25)	393	161 (40.97)	173 (44.02)	166 (42.24)

K = Rate of malaria knowledge; A = Rate of correct attitude; P = Rate of good practice.

### Comparison of knowledge, attitudes and practices (KAPs) after intervention

A comparison of the 20 major malaria-related KAP items in the test and control areas before and after the intervention yielded 14 items in which the percentage of correct or appropriate responses showed a statistically significant increase (*p* < 0.05). The general level of knowledge was higher in the test areas than in the control areas. In terms of attitudes and practices in the test areas, there was a marked increase in the avoidance of outdoor sleeping and in the conscious utilization of mosquito-protection measures (*p* < 0.05), but there were also decreases in a few items. In the indexes of the actual use of bed nets at home, accepting a blood test when having fever, and accepting ITNs at home, the percentages rose from 23.44%, 43.59%, and 34.25% to 81.08%, 85.23%, and 92.61% after the intervention, respectively, and the percentage of sleeping outdoors in the mountains decreased from 32.78% to 5.05%. These differences were statistically significant (*p* < 0.01). In the control areas, there were also increases in many indexes, but these were not significant compared with the testing area (Table 5).

**Table 5:** The proper rate of the major indexes of the malaria-related KAPs among mountain worker populations after intervention

**Index**	**Testing area**		**Control area**		***χ***^**2**^	***P***
	**n**	**%**	**n**	**%**		
How malaria is spread	473	85.20	258	65.65	49.96	0.00*
Onset symptoms of malaria	502	90.40	257	65.30	90.49	0.00*
Types of malaria	114	20.60	8	22.50	0.47	0.49
Malaria prevention methods	414	74.60	201	51.20	55.52	0.00*
Mosquito breeding grounds	490	88.20	215	54.80	136.11	0.00*
Anti-mosquito effect of insecticide treated bed nets	441	79.50	316	80.30	0.13	0.72
Whether avoiding mosquito can prevent malaria	498	89.70	290	73.80	41.66	0.00*
Using bed nets actively in the mountains	380	68.50	137	34.90	104.82	0.00*
Acceptance of information about malaria	467	84.10	344	87.50	2.14	0.14
Preventive drugs for malaria	228	41.10	155	39.50	0.26	0.61
Treatment drugs for malaria	302	54.40	179	45.60	7.24	0.01*
Method of preventive drugs	87	15.70	54	13.70	0.68	0.41
Having a plan to prevent mosquitoes	513	92.50	217	55.30	179.96	0.00*
Having a plan to not sleep outdoors in the mountains	485	87.40	264	67.20	56.67	0.00*
Taking preventive measures while working in the mountains	459	82.70	241	61.30	54.44	0.00*
Percentage sleeping outdoors in the mountains	28	5.05	105	26.70	89.60	0.01*
Actual use of a bed net at home	450	81.08	191	48.60	110.85	0.00*
Accepting a blood test when having a fever	473	85.23	274	69.60	33.11	0.00*
Accepting ITNs at home	514	92.61	219	55.80	178.53	0.00*
What to do when suffering from malaria	470	84.70	340	86.50	0.62	0.43

*p < 0.05.

Nine hundred forty-eight persons (testing area, 555; control area, 393) participated in the survey after undergoing the BBC-strategy-intervention.

### KAP summary before and after intervention

The percentages of mountain workers in the test area who passed the knowledge (K), attitudes (A), and practices (P) sections of the questionnaire increased from 37.73%, 37.00%, and 43.04% before the intervention to 89.01%, 91.53%, and 92.25% after the intervention, respectively; all three differences were statistically significant (*p* < 0.01). With gender as a categorical variable, and comparing the malaria-related KAPs of mountain workers in the test area and control area against each other or against themselves with χ2 tests, all comparisons showed statistically significant increases (*p* < 0.01; Tables 6 and 7).

**Table 6:** Malaria-related KAPs of the mountain worker populations laterally compared in different areas before and after intervention

**Gender**	**Content**	**Before intervention**						**After intervention**					
		**Testing area**		**Control area**		***χ***^**2**^	***p***	**Testing area**		**Control area**		***χ***^**2**^	***p***
		**n**	**%**	**n**	**%**			**n**	**%**	**n**	**%**		
Male	K	121	38.30	84	46.40	3.13	0.08	249	84.40	91	45.30	84.92	0.00*
	A	112	35.40	75	41.40	1.76	0.18	257	87.10	93	46.30	96.04	0.00*
	P	140	44.30	81	44.80	0.01	0.92	263	89.20	84	41.80	127.60	0.00*
Female	K	85	37.00	69	34.80	0.21	0.65	245	94.20	70	36.50	174.51	0.00*
	A	90	39.10	74	37.40	0.14	0.71	251	96.50	80	41.70	169.63	0.00*
	P	95	41.30	87	43.90	0.30	0.58	249	95.80	82	42.70	158.62	0.00*

*p *<* 0.01.

Testing area 546 persons (male, 316; female, 230) and control area 379 persons (male, 181; female, 198) participated in the survey before the intervention, testing area 555 persons (male, 295; female, 260) and control area 393 persons (male, 201; female, 192) participated in the survey after the intervention.

**Table 7:** Malaria-related KAPs of the mountain worker populations longitudinally compared in different areas before and after intervention

**Gender**	**Content**	**Testing area**						**Control area**					
		**Before intervention**		**After intervention**		***χ***^**2**^	***p***	**Before intervention**		**After intervention**		***χ***^**2**^	***p***
		**N**	**%**	**N**	**%**			**N**	**%**	**N**	**%**		
Male	K	121	38.30	249	84.40	135.84	0.00*	84	46.40	91	45.30	0.05	0.82
	A	112	35.40	257	87.10	170.33	0.00*	75	41.40	93	46.30	0.90	0.34
	P	140	44.30	263	89.20	136.67	0.00*	81	44.80	84	41.80	0.34	0.56
Female	K	85	37.00	245	94.20	182.05	0.00*	69	34.80	70	36.50	0.11	0.74
	A	90	39.10	251	96.50	190.07	0.00*	74	37.40	80	41.70	0.75	0.39
	P	95	41.30	249	95.80	173.07	0.00*	87	43.90	82	42.70	0.06	0.81

*p < 0.01.

Testing area 546 persons (male, 316; female, 230) and control area 379 persons (male, 181; female, 198) participated in the survey before the intervention, testing area 555 persons (male, 295; female, 260) and control area 393 persons (male, 201; female, 192) participated in the survey after the intervention.

### Changes in malaria serology

In the test area, the malaria IFAT positive rates fell from 4.03% before the intervention to 1.44% after the intervention; this difference was statistically significant (p < 0.01). The positive geometric mean reciprocal titer (GMRT) decreased from 27.41% to 21.81%, and the overall geometric mean reciprocal titer (GMRT) decreased from 10.42% to 10.11%. There was little change in each index in the control area, with no statistically significant differences (*p >* 0.05; Table 8).

**Table 8:** Comparison of the IFAT and blood smear test changes before and after intervention

**Group**	**Population**	**IFAT**			**GMRT**	
		**Number testing positive (%)**	***χ***^**2**^	***P***	**Positive**	**Total**
Testing area	546	22 (4.03)	6.95	< 0.01	27.41	10.42
	555	8 (1.44)			21.81	10.11
Control area	379	24 (6.33)	0.34	> 0.05	25.94	10.62
	393	21 (5.34)			24.38	10.49

IFAT = Indirect fluorescent antibody test. GMRT = Geometric mean reciprocal titer. IFAT Positive: ≥ 1:20.

### Changes in malaria infection rates

The survey results showed that, in the testing area, the percentage of subjects with blood tests revealing a parasite infection decreased from 0.18% before the BCC intervention to 0 after the intervention, the malaria cases reported (including clinical diagnoses) decreased from seven to one, and the malaria incidence rate declined from 1.71 per 10,000 (7/40,746) to 0.24 per 10,000 (1/41,244). In the control area, the blood test parasite infection rate was 0.26% before and 0.51% after the intervention; there were three malaria cases reported before and two malaria cases after the intervention. Moreover, the malaria incidence decreased from 1.31 per 10,000 (3/22,875) to 0.85 per 10,000 (2/23,541).

### Overall evaluation of the BCC intervention content

**Figure 4:**
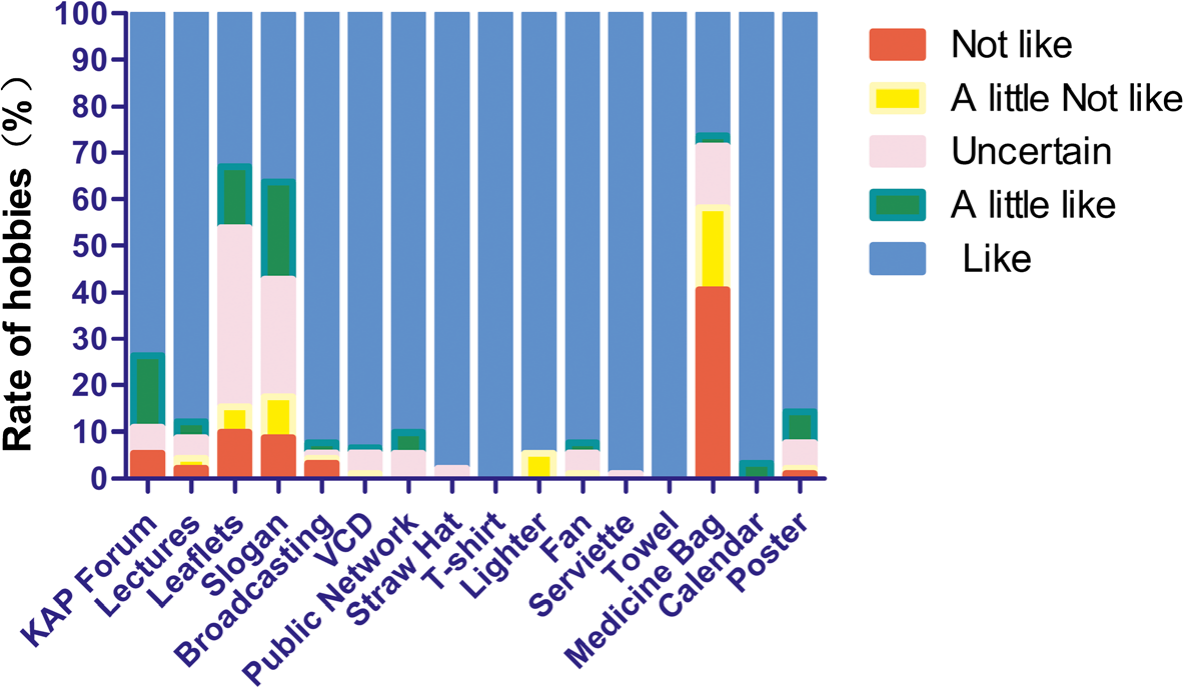
**The rate of mountain worker hobbies feedback to BCC intervention content.** *After the intervention, 100 mountain workers were randomly selected to provide feedback, 94 completed questionnaires, 91 qualified questionnaires.

During the final survey, 100 mountain workers were randomly selected to provide feedback. They were asked whether they could recall the various promotional materials and their core messages and the degree to which they liked these materials. The results showed that over 90% liked the materials (Figure 4).

## Discussion

Through the literature and this survey, that the majority of mountain workers of the Li ethnicity usually communicate in the local Hainan dialect because they cannot understand or rarely use Mandarin in the test sites. Because their customs and language are different from others and because their economic sources rely on planters of betel nuts and rubber, the residents are distributed into living in mountain settlements and temporary dormitories. In addition to the seasonal migration for planting in the local area, other migration opportunities are limited but relatively stable. In the malaria control stage, the Hainan island mountain workers population is infected with malaria. Malaria control researchers previously conducted interventions and achieved good results in the research stage [10-14,35,36]. Chen *et al.* showed that, after the intervention, the malaria-related proper KAP rate is improved; for example, the bed net use rate increased from 26.8% to 72.6%, and the annual parasite incidence (API) of malaria declined from 3.5% in 1994 to 1.1% in 1996 and 0.8% in 1997 [13]. Regarding health education, after a month of malaria knowledge intervention, the correct answer rate increased from 41.4% to 91.5% [12]. However, after the intervention, related interventions cannot continue, especially given the low rate of malaria cases, and the proper malaria prevention-related KAP rate becomes poor again in the mountain worker population. In this study, the rate of proper malaria prevention-related KAP was < 50%, and the following possible factors are considered: (1) Hainan is a tropical area, and because the weather is hot, people do not like to sleep under hanging bed nets; (2) the formal education of many mountain workers is very low, and they have an insufficient understanding of KAPs regarding malaria prevention; most of the knowledge relies on oral transmission, and after a long time, the workers will forget; (3) the mountain worker populations commonly communicate in their own dialect, and they are unaccustomed to Mandarin intervention measures; (4) certain prevention interventions are not suitable to their local festivals; (5) many mountain workers are mainly young and middle-aged, and their understanding of malaria is insufficient because they believe that health and strength can prevent malaria; and so on. Therefore, this study established that, for the core group and their members from this ethnicity, researchers at the county-level CDC, township farm village teams, and hospital and village clinic personnel, must give the participants rewards and conduct regular training to core group members 1–2 times per year in order to supervise and promote the long-term effects and to ensure that the preventive measures are as long-term and continuous as possible, as evidenced in this study of BCC strategy.

In this study, during the needs assessment for implementing BCC policies, the baseline levels of malaria-related KAP in mountain worker populations in Hainan were determined for use as a basis in developing related materials and methods. This intervention model, which correlates KAP with malaria control and prevention, has received considerable attention and promotion in the past 10 years [37], and several other countries have conducted related policies in East Asia, including Myanmar, Cambodia, and Malawi [38-40]. Of course, in addition to the above factors affecting the malaria-related KAP of residents, the mode of promotion and education, the hardware and facilities at health institutions, and social and cultural factors may also affect the effectiveness of malaria-related KAP intervention. For example, this study found that in the test sites, the majority of mountain workers of the Li ethnicity usually communicate in the Hainan language because they cannot understand or rarely use Mandarin. This suggests that when health-related educational and promotional materials and methods are developed, they should be fully integrated in the local linguistic and cultural structures. Furthermore, feedback on the BCC interventional media showed that malaria-related promotional materials should be selected based on the lifestyles and productivity of the group in question; some well-received items included straw hats, T-shirts, lighters, and fans. For audio-visual promotional materials such as VCDs and public broadcasting, including Hainan minority language features (such as using Hainan and Li dialects) and images and sounds of local customs (such as local festivals) made the materials more acceptable and likable. Otherwise, the implementation of the anti-malaria measures may be affected, and the desired goals may not be reached. These results are in agreement with the results of similar research conducted in rural areas of Hubei, China [41]. Regarding other aspects of malaria serology, the BCC strategy was found to be statistically significant (*P* < 0.01) through a comparison of the IFAT and blood smear test changes before and after the intervention. Furthermore, the positive and overall GMRTs shows little change because all of the malaria-control measures are better in the testing and control areas; however, the BCC strategy is more effective than the conventional measures [33].

The survey results showed that the mountain workers in Hainan had a better understanding of malarial symptoms and transmission pathways than did the people in the rural areas of Hubei, China [42,43]. One possible explanation is that the mountain workers were mainly planters who must go into the mountains frequently and thus have a higher risk of infection. The aim of the BCC intervention in this study was to improve the ability of the mountain workers themselves to prevent malaria, especially by seeking medical care promptly, using appropriate medication, and utilizing ITNs and LLTNs [44-47]. In the test sites, the majority of mountain workers showed significant improvement in malaria-related KAP indices after intervention; this finding is similar to the results of studies conducted in Malaysia, Bhutan, Vietnam, and Swaziland [48-51]. In contrast, although the living conditions in areas with concentrated ethnic minority populations have gradually improved in the past few years, and mountain houses and straw huts are decreasing in number due to the government’s efforts to improve dangerous housing conditions, these surveys showed that mosquito protection measures, such as household bed nets and screen windows, are rarely used, and the use of preventative medication and night-time mosquito protection when going into the mountains is not ideal either. Related studies suggest the importance of mosquito protection in living quarters [52,53].

In this study, implementing BCC policies through mountain-worker community participation achieved good results. Malaria prevention core groups were established, consisting of county-level CDC, township farm village teams, and hospitals and village clinic personnel; strengthened the community outreach mobilization; implemented periodic coordination and training on routine handling procedures upon discovery of malaria; expanded the core group members by influencing the family members and coworkers of mountain workers in the region; and used a malaria reporting reward system to ensure that the policies were implemented. The study also relied on the public health broadcasting system, which the Hainan government had installed in health care facilities, community health service stations, urban and rural hospitals, pharmacies and other public places. Malaria prevention facts were also put on rolling display every day on LCD digital media platforms, and the intervention was effective. These results are similar to those achieved by implementing BCC policies to promote malaria prevention KAPs in the South Pacific island of Vanuatu and in malaria-endemic areas of India [53,54]. At the same time, studies also suggest that when promoting health education, the positive role played by women and family members in community interventions must be emphasized [55,56].

China’s action plan to eliminate malaria (2010–2020) set up a specific timetable for the elimination of malaria. Developing materials and methods according to local conditions for malaria-related interventions using comprehensive BCC models can provide both a theoretical basis and practical methods for eliminating malaria in Hainan. However, this study has certain limitations: the positive malaria antibody cases in the present study were not necessarily actual malaria cases, malaria itself has more complex epidemic factors, this study only selected representative factors among the various factors of KAP, and misunderstanding by the survey subjects might have affected the investigation. The present investigation does, however, provide a scientific basis and theoretical reference for employing the BCC strategy to eliminate malaria in Hainan.

## Competing interests

The authors declare that they have no competing interests.

## Authors’ contributions

HCH, DJW, WSQ conceived this study and drafted the first version of this manuscripts. HXM and WGZ gave technical assists. ZW, SDW, CCX participated the survey and data collection. LYC polished and adjusted figures and tables in this manuscripts. All of the authors have read and approved the final manuscript.
